# Effect of incubation temperature and substrate moisture on embryonic development, hatchling phenotypes and post-hatching growth in the Reeves’ Turtle, *Mauremys reevesii*

**DOI:** 10.7717/peerj.10553

**Published:** 2021-02-12

**Authors:** Yufeng Wei, Yangchun Gao, Dainan Cao, Yan Ge, Haitao Shi, Shiping Gong

**Affiliations:** 1 Guangdong Key Laboratory of Animal Conservation and Resource Utilization, Guangdong Public Laboratory of Wild Animal Conservation and Utilization, Institute of Zoology, Guangdong Academy of Sciences, Guangzhou, China; 2 Ministry of Education Key Laboratory for Ecology of Tropical Islands, College of Life Sciences, Hainan Normal University, Haikou, China

**Keywords:** *Mauremys reevesii*, Incubation temperature, Substrate moisture, Hatchling phenotypes, Post-hatching growth

## Abstract

**Background:**

Reeves’ Turtles (*Mauremys reevesii*) are economically important in aquaculture in China. Understanding the effects of incubation temperature and substrate moisture on embryos and hatchlings is of great significance for improving the artificial culture of *M. reevesii*. However, available studies have not yet determined the thermal and hydric optima for *M. reevesii* eggs, and the potential interaction between the two factors.

**Methods:**

In this study, eggs of *M. reevesii* were incubated at five temperature levels (23, 26, 29, 32 and 35 °C, fluctuation range ± 0.5 °C). In each temperature level, there were three substrate moisture levels (1:0.5, 1:0.9 and 1:1.2, weight ratio of vermiculite to water). Thus, a total of 15 combinations of temperature and moisture were used to examine the effects of incubation temperature and substrate moisture on incubation duration, hatching success, hatchling phenotypes, post-hatching growth and hatchling survival.

**Results:**

Substrate moisture did not significantly affect most development parameters (except incubation duration and carapace width of hatchlings). Eggs incubated at low moisture level (1:0.5) had a longer incubation duration and produced hatchlings with smaller carapace widths than those incubated at medium (1:0.9) or high (1:1.2) moisture levels. Incubation temperature had a significant effect on incubation duration, hatching success, hatchling phenotypes and hatchling survival. Incubation duration decreased as incubation temperature increased. Eggs incubated at 23, 26 and 29 °C showed higher hatching success than those incubated at 32 and 35 °C. Hatchlings incubated at 32 °C were smaller in body size and mass than those incubated at 23, 26 and 29 °C. At 12 months of age, incubation temperature had no long-lasting effect on body mass, but hatchlings incubated at 23 and 35 °C had lower survival rates than those incubated at 26, 29 and 32 °C. For the development of embryos and hatchlings, the interaction between incubation temperature and substrate moisture was not significant.

**Conclusions:**

Our results indicate that incubation temperature has a significant influence on the development of embryos and hatchlings of *M. reevesii*, while substrate moisture only significantly affects the incubation duration and carapace width of hatchlings. The combination of an incubation temperature of 29 ± 0.5 °C and a substrate moisture level of 1:1.2 represented optimal incubation conditions in this experiment. Such incubation conditions are helpful in obtaining higher hatching success, shorter incubation duration and higher survival rates for this aquaculture species.

## Introduction

Freshwater aquaculture has grown rapidly over the last three decades in China (Wang et al., 2015). Optimising incubation conditions is crucial to improve hatching success, post-hatching growth and survival for aquaculture species, especially for turtles (Du & Ji, 2003; Zhu et al., 2006). Turtle egg incubation is influenced by many environmental factors, of which temperature and moisture are the most important (Packard, 1999; Delmas et al., 2008). Incubation temperatures have significant effects on incubation duration, hatching success, hatchling phenotypes, and post-hatching growth in turtles (Janzen & Morjan, 2002; Du & Ji, 2003; Du et al., 2007). Specifically, some studies found that hatchlings incubated at low temperatures grew faster in post-hatching growth experiments than did those incubated at high temperatures (Du & Ji, 2003; Ji et al., 2003). However, the opposite result was also frequently reported in turtles (Janzen & Morjan, 2002; Du et al., 2007). In contrast, there was one study showing that hatchlings incubated at moderate temperatures grew faster (Brooks et al., 1991). Hence, the influence of incubation temperature on embryonic development and subsequent growth is still unclear.

In addition to incubation temperature, substrate moisture also has significant effects on incubation duration, hatching success, and hatchling phenotypes in turtles (Packard, 1999; Tucker & Paukstis, 2000). Moisture has varying influences on turtles depending on eggshell type (i.e., flexible-shelled eggs or rigid-shelled eggs; see Booth, 2002). Flexible-shelled eggs incubated at high moisture levels have longer incubation duration, higher hatching success, and produce larger hatchlings in comparison to those incubated at low moisture levels (Tucker et al., 1998; Packard, 1999; Tucker & Paukstis, 2000). In contrast, for rigid-shelled eggs, moisture has no significant effect on incubation duration and hatching success (Booth, 2002; Zhao et al., 2009). Eggs incubated at low moisture levels produce lighter hatchlings with a larger residual yolk mass than those incubated at high moisture levels, which may have long-term consequences with regard to growth and survival after hatching (Finkler, Knickerbocker & Claussen, 2000; Booth, 2002). However, such long-term experiments have only been carried out in a few species (Brooks et al., 1991; Miller, 1993; Bobyn & Brooks, 1994), and the influence of substrate moisture on incubation duration, hatching success, and hatchling phenotypes is unknown for many turtle species.

In China, the wild population of Reeves’ Turtles (*Mauremys reevesii*) is endangered (Jiang et al., 2016), but the species is extensively cultured due to its high commercial value (Du et al., 2007; Shi et al., 2008). However, only five rigid-shelled eggs on average are laid per clutch (Liu, Xu & Liu, 1988). Therefore, it is very important to determine optimal incubation conditions. Previous investigations indicated that incubation temperature has significant effect on incubation duration, hatching success, hatchling phenotypes, and growth rate (Du et al., 2007; Wang et al., 2010), but the effect of substrate moisture on embryos and hatchlings has only been investigated at a single temperature (Du & Zheng, 2004). There may also be interactive effects of incubation temperature and substrate moisture that ultimately affect embryonic growth rates or survival (Packard et al., 1987), but no study on *M. reevesii* has been undertaken. In addition, the possible long-term effects of incubation temperature and substrate moisture remain unclear in previous studies. In particular, post-hatching growth and survival is an issue of importance in the artificial culture of turtles. In this study, eggs of *M. reevesii* were incubated at five incubation temperatures and three substrate moisture levels to understand the effects of both factors on incubation duration, hatching success, hatchling phenotypes, post-hatching growth and hatchling survival. This study will provide important information for the artificial culture and biological conservation of this species.

## Materials and Methods

### Egg collection and screen

Eggs of *M. reevesii* were collected from Guangdong Lvca Industry Co., Ltd. (Boluo Guangdong, China) in early June, 2017. All eggs were laid within a 24 h period to ensure consistent embryo ages. Eggs were temporarily stored in a foam box full of moist vermiculite, and transferred to our laboratory at Guangzhou. It is not easy to determine whether newly laid eggs are fertilized (Liu et al., 1984). Thus we only removed damaged or deformed eggs by visual inspection, and 450 eggs were retained and used for the present study. The eggs were numbered on the shell using a pencil, and weighed to the nearest 0.1 g using an electronic balance (Shanghai Huachao Electric Appliance Co., Ltd, Shanghai, China). We could not identify the parents of these eggs, but all 450 eggs came from about 90 clutches according to the average clutch size (five eggs) in this species (Liu, Xu & Liu, 1988). The different clutch origins and random allocation of eggs among different incubation conditions were expected to minimise the effects of inter-clutch differences (Du, Zheng & Shu, 2006).

### Temperature and moisture conditions of incubation

Eggs were randomly allocated into 45 plastic containers (170 mm × 116 mm × 70 mm) with vermiculite as an incubation substrate (10 eggs per container). The containers were randomly allocated to five incubators (Tianjin City Taisite Instrument Co., Ltd, Tianjin, China), so there were nine containers per incubator. The incubator temperatures were set at 23, 26, 29, 32 and 35 °C (fluctuation range ±0.5 °C), respectively. Two digital thermometers (Changzhou Ruiming Instrument and Meter Plant Thermometer, Jiangsu, China) were put into each incubator to monitor real-time temperature and avoid errors caused by instrument failure. In each temperature level, there were three substrate moisture levels (weight ratio of vermiculite to water), including 1:0.5 (low), 1:0.9 (medium), and 1:1.2 (high), so there were three containers per moisture level. After 2 days of incubation, a white spot on the eggshell surface indicated eggs were fertilized (Liu et al., 1984; Du, Zheng & Shu, 2006). Any eggs without the white spot were removed, as they were unfertilized or dead. Every second day, we removed the eggs from the containers and added isothermal water (stored in advance in each incubator) to the substrate until it regained its initial weight, aiming to maintain the initial moisture level.

### Hatchling phenotypes

Upon hatching, the incubation duration, hatching success rates and hatchling malformation rates (including blindness, lack of tail, wry mouth and deformation of carapace or plastron) were calculated. Each hatchling was weighed (initial body mass) using an electronic balance, measured (carapace length, width and height) using a vernier caliper (Mitutoyo, Sakado, Japan) (Xiao, Shi & Sun, 2014), and marked individually by scute cutting for future identification. After measurement, the hatchlings were put into 15 plastic containers (300 mm × 243 mm × 100 mm) according to the different temperature and moisture conditions applied during incubation. The breeding room temperature ranged from 19.1 to 29.6 °C during the experiment. Every second day, the hatchlings were fed with an appropriate commercial diet (Shenzhen Inch-Gold Fish Food Co., Ltd, Guangdong, China). The body mass of each turtle was measured at 1, 2, 3 and 12 months after hatching. All experimental procedures were performed in accordance with the regulations of the Animal Care and Welfare Committee, Guangdong Institute of Applied Biological Resources (GIABR20170601).

### Statistical analysis

Statistical analysis was mainly done using SPSS 25.0. We used logistic regression to investigate the effects of temperature, moisture and egg mass on hatching success, hatchling malformation rate and survival rate. The categorical variable was assigned a value of 0 or 1, indicating hatched or unhatched, malformed or normal, death or survival of hatchling turtles, respectively. For continuous variables, we used linear regression to examine the effects of temperature, moisture and egg mass on incubation duration, hatchling phenotypes and post-hatching growth. Given only four hatchlings were detected from eggs incubated at 35 °C, and all these hatchlings were malformed and died within the first 7 days after hatching (Table 1), the data from these individuals was only used to analyze the difference in hatching success. When variables were simultaneously affected by two or more factors, variance partitioning was performed to estimate the explained percentage of those factors on variables using the *vegan* package implemented in R. The statistical data were presented as mean ± standard deviation (SD) and the significance level was set at *p* < 0.01.

**Table 1:** The effects of incubation temperature and substrate moisture on incubation duration, hatching success, hatchling malformation rates and survival rates in *Mauremys reevesii*.

Incubation temperature (°C)	Substrate moisture	No. of fertilized eggs	Hatching success (%)	Malformation rate of hatchlings (%)	Survival rate of hatchlings (%)	Incubation duration (range)
23	1:0.5	19	73.7 (14/19)	21.4 (3/14)	35.7 (5/14)	117.1 ± 2.4 (115.0∼121.0)
	1:0.9	22	81.8 (18/22)	22.2 (4/18)	66.7 (12/18)	113.7 ± 3.9 (107.0∼119.0)
	1:1.2	18	72.2 (13/18)	23.1 (3/13)	61.5 (8/13)	113.7± 2.9 (109.0∼117.0)
26	1:0.5	19	100.0 (19/19)	10.5 (2/19)	94.7 (18/19)	86.3 ± 3.2 (82.0∼92.0)
	1:0.9	16	87.5 (14/16)	28.6 (4/14)	92.9 (13/14)	83.9 ± 2.5 (79.0∼88.0)
	1:1.2	23	95.7 (22/23)	13.6 (3/22)	90.9 (20/22)	84.8 ± 2.5 (80.0∼88.0)
29	1:0.5	20	80.0 (16/20)	12.5 (2/16)	100.0 (16/16)	72.1 ± 1.8 (70.0∼75.0)
	1:0.9	27	85.2 (23/27)	8.7 (2/23)	100.0 (23/23)	71.7 ± 2.4 (68.0∼75.0)
	1:1.2	30	96.7 (29/30)	3.4 (1/29)	89.7 (26/29)	68.9 ± 3.3 (63.0∼73.0)
32	1:0.5	27	48.1 (13/27)	15.4 (2/13)	92.3 (12/13)	62.2 ± 2.4 (59.0∼66.0)
	1:0.9	26	53.8 (14/26)	0 (0/14)	85.7 (12/14)	59.5 ± 2.4 (56.0∼63.0)
	1:1.2	23	34.8 (8/23)	12.5 (1/8)	87.5 (7/8)	60.0 ± 2.3 (56.0∼63.0)
35	1:0.5	23	0 (0/23)	−	−	−
	1:0.9	24	4.2 (1/24)	100.0 (1/1)	0 (0/1)	−
	1:1.2	20	15.0 (3/20)	100.0 (3/3)	0 (0/3)	−

**Note:**

“−” indicates no data. Survival rates of hatchlings were calculated at 12 months of age.

## Results

### Effects of incubation temperature and substrate moisture on embryonic development

After 2 days’ incubation, we removed the unfertilized eggs from each treatment group. The number of fertilized eggs, hatching success, hatchling malformation rate and incubation duration in each treatment group are shown in Table 1. The logistic regression analysis showed that substrate moisture (β = 0.026, *p* = 0.962) and egg mass (β = −0.071, *p* = 0.664) had no significant effect on hatching success. In contrast, incubation temperature significantly affected hatching success (β = −1.162, *p* < 0.0001). Eggs incubated at 23, 26 and 29 °C had higher hatching success than those incubated at 32 and 35 °C (Table 1). Within the range from 23 to 32 °C, hatchling malformation rate was not significantly affected by incubation temperature (β = 0.045, *p* = 0.935), substrate moisture (β = −0.395, *p* = 0.531) and egg mass (β = 0.351, *p* = 0.210). The linear regression analysis showed that incubation temperature (β = −18.083, *p* < 0.0001) and substrate moisture (β = −1.925, *p* < 0.0001) significantly affected incubation duration. Incubation duration decreased as incubation temperature or substrate moisture increased (Table 1). In contrast, egg mass had no significant effect on incubation duration (β = −0.042, *p* = 0.935).

### Effects of incubation temperature and substrate moisture on hatchling phenotypes

Incubation temperature significantly affected hatchling phenotypes (body mass (BM: β = −0.107, *p* < 0.0001); carapace width (CW: β = −0.261, *p* = 0.001); carapace height (CH: β = −0.352, *p* < 0.0001); but not carapace length (CL: β = −0.097, *p* = 0.163)). BM, CW and CH of hatchlings decreased as incubation temperature increased. Hatchlings from eggs incubated at 32 °C were smaller and lighter compared to those incubated at other temperatures (Table 2). In contrast, substrate moisture had no significant effect on hatchling phenotypes (BM: β = 0.039, *p* = 0.184; CL: β = 0.005, *p* = 0.950; CH: β = 0.001, *p* = 0.991), except CW (β = 0.272, *p* = 0.004). CW of hatchlings increased as substrate moisture increased (Table 2). Egg mass had a significant effect on hatchling phenotypes (BM: β = 0.514, *p* < 0.0001; CL: β = 1.348, *p* < 0.0001; CW: β = 0.980, *p* < 0.0001; CH: β = 0.518, *p* < 0.0001).

**Table 2:** Descriptive statistics for initial body mass (g), carapace length, width and height (mm) of hatchlings at different incubation temperature and substrate moisture in *Mauremys reevesii*.

Incubation temperature (°C)	Substrate moisture	No. of hatchlings	Initial body mass (range)	Carapace length (range)	Carapace width (range)	Carapace height (range)
23	1:0.5	14	3.5 ± 0.5 (2.4∼4.2)	23.28 ± 1.13 (21.00∼24.62)	17.57 ± 0.93 (15.90∼19.38)	13.39 ± 0.78 (12.32∼14.66)
	1:0.9	18	3.7 ± 0.6 (2.8∼4.9)	23.37 ± 1.27 (20.30∼26.16)	19.33 ± 1.52 (16.40∼22.70)	13.56 ± 0.88 (12.16∼15.34)
	1:1.2	13	3.8 ± 0.5 (2.9∼4.6)	23.45 ± 1.23 (21.00∼25.00)	19.66 ± 1.04 (18.16∼21.56)	13.89 ± 0.77 (12.34∼15.00)
26	1:0.5	19	3.7 ± 0.5 (2.4∼4.3)	24.11 ± 1.51 (20.68∼25.86)	19.47 ± 1.13 (16.18∼21.12)	13.58 ± 0.68 (11.88∼14.76)
	1:0.9	14	3.4 ± 0.4 (2.7∼3.9)	23.39 ± 1.03 (21.18∼24.66)	18.90 ± 0.80 (17.66∼20.60)	13.21 ± 0.59 (11.88∼13.98)
	1:1.2	22	3.6 ± 0.5 (2.7∼4.8)	23.68 ± 1.22 (21.18∼26.40)	19.52 ± 1.24 (16.64∼22.00)	13.30 ± 0.57 (12.10∼14.44)
29	1:0.5	16	3.4 ± 0.8 (2.0∼4.6)	23.18 ± 2.44 (18.88∼26.92)	18.78 ± 1.42 (15.68∼20.64)	12.99 ± 0.91 (11.46∼14.60)
	1:0.9	23	3.4 ± 0.5 (2.4∼4.3)	23.61 ± 1.23 (21.18∼25.70)	19.13 ± 1.15 (16.66∼20.90)	12.83 ± 0.56 (11.40∼13.86)
	1:1.2	29	3.5 ± 0.5 (2.8∼4.8)	23.60 ± 1.54 (20.72∼27.00)	19.09 ± 1.39 (14.96∼21.66)	12.90 ± 0.69 (11.82∼14.48)
32	1:0.5	13	3.2 ± 0.3 (2.6∼3.9)	22.69 ± 0.97 (21.12∼24.26)	17.89 ± 0.69 (16.92∼19.24)	12.37 ± 0.52 (11.48∼13.36)
	1:0.9	14	3.1 ± 0.5 (2.4∼3.8)	22.27 ± 1.35 (20.10∼24.52)	17.26 ± 1.16 (15.40∼19.32)	12.29 ± 0.81 (11.00∼13.56)
	1:1.2	8	3.4 ± 0.4 (2.6∼3.9)	22.81 ± 1.25 (20.80∼24.08)	17.69 ± 1.08 (15.46∼18.90)	12.66 ± 0.88 (12.00∼14.66)

### Effects of incubation temperature and substrate moisture on post-hatching survival and growth

**Figure 1:**
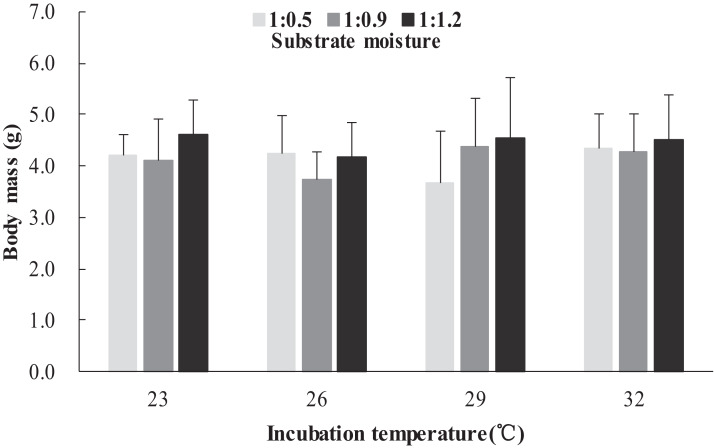
Body mass of hatchlings incubated from different incubation temperature and substrate moisture at 1 month of age in *Mauremys reevesii*.

**Figure 2:**
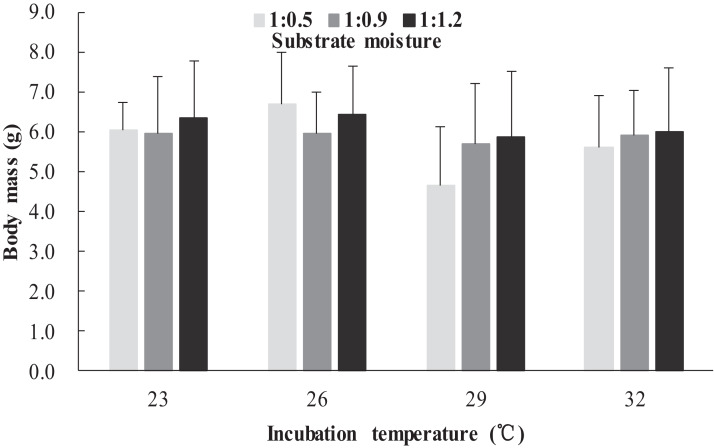
Body mass of hatchlings incubated from different incubation temperature and substrate moisture at 2 month of age in *Mauremys reevesii*.

**Figure 3:**
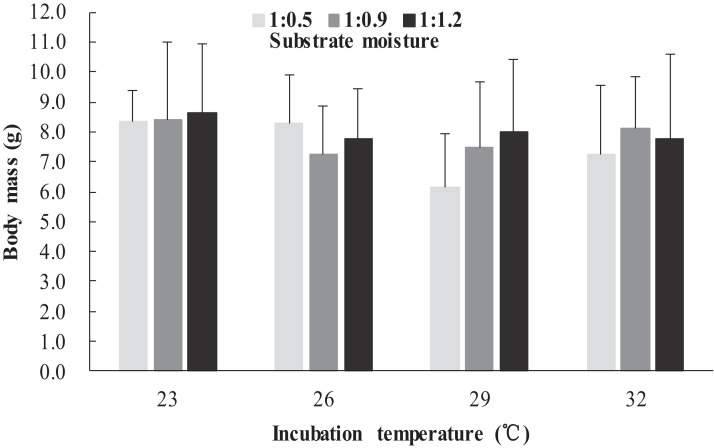
Body mass of hatchlings incubated from different incubation temperature and substrate moisture at 3 month of age in *Mauremys reevesii*.

**Figure 4:**
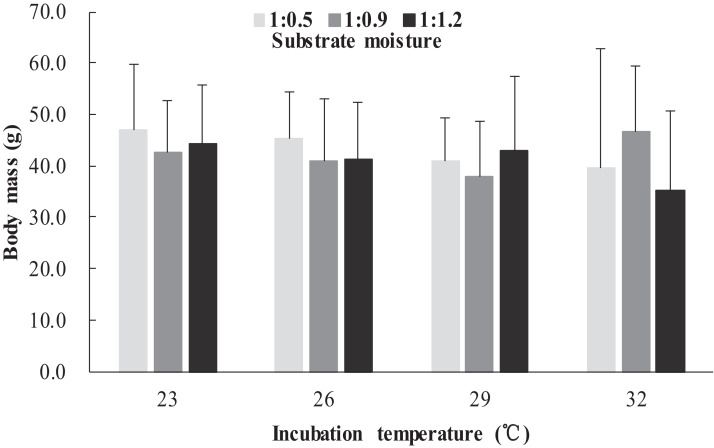
Body mass of hatchlings incubated from different incubation temperature and substrate moisture at 12 month of age in *Mauremys reevesii*.

The survival rates of hatchlings from different treatment groups at 12 months of age are shown in Table 1. Logistic regression analysis showed that substrate moisture (β = 1.109, *p* = 0.085) and egg mass (β = 0.391, *p* = 0.173) had no significant effects on survival rates, whereas incubation temperature significantly affected survival rates (β = 2.210, *p* = 0.004). Hatchlings from eggs incubated at 26, 29 and 32 °C had higher survival rates than those incubated at 23 °C (Table 1). During the growth experiment, we found that incubation temperature and substrate moisture had no significant effects on the turtles’ mass at any stage (*p* > 0.05, in all cases). The body masses of turtles from the different treatment groups are shown in Figs. 1–4. In contrast, egg mass significantly affected the turtles’ mass at 1 (β = 0.667, *p* < 0.0001), 2 (β = 0.878, *p* < 0.0001) and 3 (β = 1.011, *p* < 0.0001) months of age, but not at 12 months of age (β = 2.136, *p* = 0.090).

### Explanatory variables for embryonic development parameters

**Figure 5:**
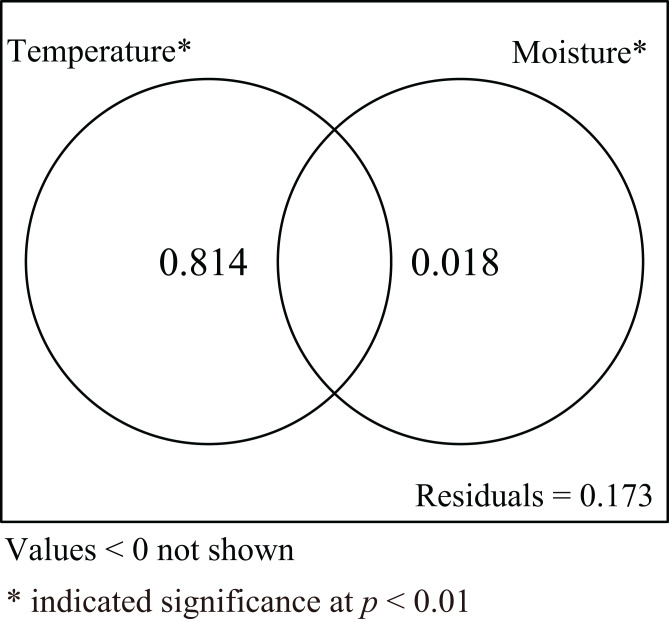
Venn diagram for two explanatory variables. The results of variance partitioning analysis to assess the response of incubation temperature and substrate moisture to incubation duration and carapace width of hatchling turtles.

Incubation duration and CW of hatchlings were influenced by both incubation temperature and substrate moisture. To explore the relative roles of incubation temperature and substrate moisture on those parameters, variance partitioning was performed for explanatory variables. The results showed that incubation temperature alone explained 81.4% of the total variation when the influence of moisture was excluded. Conversely, substrate moisture alone explained 1.8% of total variation when the influence of temperature was removed. The shared explained percentage between incubation temperature and substrate moisture was less than 0, meaning the interaction between incubation temperature and substrate moisture was not significant (Fig. 5).

## Discussion

### Effect of incubation temperature on embryos and hatchlings

Incubation temperature significantly affected hatchling phenotypes, with smaller and lighter hatchlings being produced at high temperature (32 °C) than at lower temperatures (23, 26 and 29 °C). In previous studies, there have been many examples of incubation temperature influencing hatchling phenotypes in turtles. For example, in a recent study by Stubbs & Mitchell (2018) on *Chelonia mydas*, hatchlings from eggs incubated at 31 °C were smaller and had greater residual yolk mass than those incubated at 27 °C. The degree of yolk absorption at different incubation temperatures may be one of the important factors affecting hatchling phenotypes. At high temperatures, embryonic growth is faster and incubation time is shorter, meaning the hatchlings have less time to convert the yolk into body tissue before pipping, thus producing smaller hatchlings and greater residual yolk mass (Ji & Zhang, 2001). This was also supported by the observation that residual yolk mass increased with temperature over a temperature range from 24 to 33 °C in *M. reevesii* (Zheng et al., 2006). Thus, we speculated that the differences in hatchling phenotypes may be related to the residual yolk mass in *M. reevesii*. In addition, it is noteworthy that differences in hatchling phenotypes at different incubation temperatures may involve confounding effects of temperature and hatchling sex, since *M. reevesii* is a temperature-dependent sex determination species (Hou, 1985). For example, evidence shows that there is no significant difference in hatchling phenotypes between different temperatures within the range of males’ incubation temperatures (less than 27 °C), or within the range of females’ incubation temperatures (more than 30 °C) (Du et al., 2007). More specifically, hatchling phenotypes showed no significant difference between 24 and 26 °C or between 30 and 32 °C (Du et al., 2007). However, the molecular mechanism of incubation temperatures’ effects on hatchling phenotypes and whether the hatchling phenotype differences are related to hatchling sex remain unknown and need further study.

During the growth experiment, we found that incubation temperatures had no long-lasting impact on body mass. Similar results have been reported by Du et al. (2007), who found no significant difference in body mass between different incubation temperatures when the turtles were 3 months old, suggesting that the small hatchlings incubated at high temperatures may catch up with larger individuals incubated at low temperatures during subsequent growth, when provided with suitable environments (e.g. unlimited food, suitable temperatures and no predators). The growth rate and mass of females incubated at high temperatures were higher than those of males incubated at low temperature at 6 and 9 months of age (Du et al., 2007). The intersexual variation in hatchling growth was closely related to sexual size dimorphism, with the larger sex (female *M. reevesii*) having a greater growth rate (Du et al., 2007; Du & Shen, 2007). However, differences in growth rates between sexes were not observed in this study. It is likely that the differences between studies were due to the temperatures at which hatchlings and juveniles were reared. While we cultured our hatchlings and juveniles turtles at room temperature (19.1–29.6 °C), Du et al. (2007) used a high and constant temperature (30 ± 1 °C). Growth rates of hatchling *M. reevesii* at constant temperature (30 °C) are significantly higher than those at variable temperatures (7.5–30 °C) (Li, 2005). It is speculated that greater growth rates in high and constant temperatures may accelerate the differentiation of sexual dimorphism.

The survival rates of hatchlings incubated at 23 °C (55.6%) and 35 °C (0%) were lower than those incubated at 26 °C (92.7%), 29 °C (95.6%) and 32 °C (88.6%) over 12 months (Table 1). This is mainly because hatchlings incubated at intermediate temperatures may have higher fitness, which can be influenced by different energy availability between high and low incubation temperatures. More specifically, longer incubation duration at low temperatures leads to increased embryonic energy expenditure, and longer incubation times also mean hatchlings have had shorter growth periods before the first winter comes. This may result in reduced hatchling survival (Du & Ji, 2001; Zheng et al., 2006). Under high temperatures, higher embryonic metabolic rates lead to increased energy consumption (Du & Ji, 2001). High temperatures also lead to increased hatchling malformation rates, and those individuals usually die in aquaculture (Zhu et al., 2006). In contrast, eggs incubated at intermediate temperatures not only transferred more dry matter, lipids and energy from the egg yolk into the body and tissue than those incubated at extremely high and low temperatures, but hatchlings also showed better locomotor performance (Du & Ji, 2001; Du, Zheng & Shu, 2006; Zheng et al., 2006). Therefore, incubation temperature may indirectly affect hatchling survival by influencing their energy conversion and locomotor performance.

The optimum temperature and the upper limit temperature of embryonic development are two of the key concerns for the aquaculture industry and biological conservation. A number of studies revealed noticeable interspecific differences in the optimum temperature and the upper limit temperature of embryonic development (Yang, Niu & Sun, 2002; Zhu et al., 2006; Du et al., 2007; Fordham, Georges & Corey, 2007; Du, Wang & Shen, 2010; Hernandez-Montoya, Paez & Ceballos, 2017). Our study indicates that the optimum temperature is 29 °C, which is similar to that for turtles such as *Mauremys mutica* (Zhu et al., 2006). In contrast, some other turtles have relatively low optimum temperatures (e.g., 28 °C); for example, *Chelodina rugosa* (Fordham, Georges & Corey, 2007) and *Mauremys sinensis* (Du, Wang & Shen, 2010). The optimum temperature for *Pelodiscus sinensis* is 32 °C (Yang, Niu & Sun, 2002). For the upper limit temperature in the current study, we found that only about half of the individuals pipped (the average hatching success was 45.6%) when the incubation temperature reached or exceeded 32 °C, suggesting that an incubation temperature of 32 °C is harmful to embryonic development and may be close to the upper limit for embryonic development in *M. reevesii*. In contrast, some other turtles have relatively high upper temperature limits, in the range of 33–34 °C, such as *M. mutica* (Zhu et al., 2006) and *P. sinensis* (Yang, Niu & Sun, 2002). Conversely, the upper limit temperature of *Chelonoidis carbonarius* is 29 °C (Hernandez-Montoya, Paez & Ceballos, 2017). Due to interspecific variations in optimum temperature and upper temperature limits for embryonic development, it is necessary to provide optimum management recommendations according to the characteristics of the species in aquaculture and conservation.

### Effect of substrate moisture on embryos and hatchlings

Our study indicated that substrate moisture had no significant effect on hatching success, in agreement with previous studies on rigid-shelled eggs (Booth, 2002; Du & Zheng, 2004; Booth & Yu, 2009; Zhao et al., 2013). Conversely, in flexible-shelled eggs, hatching success decreases as substrate moisture decreases (Packard et al., 1987; Tucker & Paukstis, 2000; Hewavisenthi & Parmenter, 2001). Variations in hatching success at different substrate moisture levels may be closely related to eggshell type. The outer layer of flexible-shelled eggs is lightly calcified, making it relatively easy for eggs to swell or shrink, whereas rigid-shelled eggs have a heavily calcified outer layer that may hinder swelling or shrinking (Packard, 1999). Compared with flexible-shelled eggs, rigid-shelled eggs have a reduced capacity for exchanging water with external environments; water is less easily lost with decreasing substrate moisture levels, and embryos show higher tolerance to variations in substrate water content (Packard et al., 1981; Packard, 1999). For this reason, substrate moisture variation may not have a significant influence on hatching success for rigid-shelled eggs.

A large number of studies have shown that flexible-shelled eggs incubated at high moisture levels have longer incubation durations than those incubated at low moisture levels (Tucker et al., 1998; Packard, 1999; Tucker & Paukstis, 2000). A study in turtles with rigid-shelled eggs also found a similar phenomenon (Zhao et al., 2013). The longer incubation duration at high moisture levels may be associated with a slower development rate of the embryos (Du et al., 2010). However, there is one contrary opinion, indicating that embryos in high moisture conditions have faster growth rates and metabolic rates (Packard, 1999). In the present study, we found that incubation duration decreased as substrate moisture increased. For example, for eggs incubated at 29 °C the incubation period at the high moisture level (1:1.2) was shorter by 3.2 d and 2.8 d than that at low (1:0.5) and medium (1:0.9) moisture levels, respectively (Table 1). Similarly, in a study by Zhao et al. (2013) on *P. sinensis*, for eggs incubated at 28 °C the incubation period at a moisture level of 1:1 was shorter by 1.6 d than that at a moisture level of 1:0.38. Evidence shows that embryonic development is inhibited under low moisture levels (Packard & Packard, 2002), and this may be one of the important reasons for the longer incubation period.

The influence of substrate moisture on embryonic development in turtles largely depends on the water exchange between the eggs and the environment, which is mainly affected by incubation temperature, substrate type, eggshell type, egg size and the contact area between the egg and its substrate (Packard, 1999; Booth, 2002). Of all the factors that affect water exchange, incubation temperature is probably the most important. A study by Packard et al. (1987) on *Chelydra serpentina* found that the interaction between incubation temperature and substrate moisture significantly affected embryonic development. In contrast, our study indicated that the interaction between incubation temperature and substrate moisture had no significant effect on the development of embryos and hatchlings (Fig. 5). There may two reasons for this result: (1) rigid-shelled eggs have a reduced capacity for exchanging water with external environments, resulting in an insignificant effect of moisture levels on embryonic development (Packard et al., 1981; Packard, 1999); and (2) perhaps because of the small range of substrate moisture we used in the study, the effect of incubation temperature may have masked the potential interaction between temperature and moisture. Further studies with larger moisture ranges and different eggshell types may provide comprehensive insights into the interactive effect of substrate moisture and incubation temperature on embryonic development in turtles.

## Conclusions

In conclusion, the results showed that incubation temperature significantly affected most development parameters (including incubation duration, hatching success, hatchling phenotypes and post-hatching survival), while substrate moisture only had a significant influence on the incubation duration and carapace width of hatchlings. A temperature of 29 ± 0.5 °C and a moisture level of 1:1.2 (weight ratio of vermiculite to water) were optimal combination conditions for egg incubation in this experiment, because eggs incubated at the optimal conditions had high hatching success, high survival rates and short incubation duration.

## Supplemental Information

10.7717/peerj.10553/supp-1 Supplemental Information 1 The raw measurements of incubation duration, hatching success, hatchling malformations, survival rates of hatchlings, hatchling phenotypes, post-hatching growth and daily weight gain of hatchlings.

## Additional Information and Declarations

### Data Availability

The following information was supplied regarding data availability: Raw data, including incubation duration, hatching success, hatchling malformations, survival rates of hatchlings, hatchling phenotypes, post-hatching growth and daily weight gain of hatchlings, are available as a Supplemental File.

## References

[ref-1] Bobyn ML, Brooks RJ (1994). Interclutch and interpopulation variation in the effects of incubation conditions on sex, survival and growth of hatchling turtles (*Chelydra serpentina*). Journal of Zoology.

[ref-2] Booth DT (2002). Incubation of rigid-shelled turtle eggs: do hydric conditions matter?. Journal of Comparative Physiology B: Biochemical, Systemic, and Environmental Physiology.

[ref-3] Booth DT, Yu CY (2009). Influence of the hydric environment on water exchange and hatchlings of rigid-shelled turtle eggs. Physiological and Biochemical Zoology.

[ref-4] Brooks RJ, Bobyn ML, Galbraith DA, Layfield JA, Nancekivell EG (1991). Maternal and environmental influences on growth and survival of embryonic and hatchling snapping turtles (*Chelydra serpentina*). Canadian Journal of Zoology.

[ref-5] Delmas V, Bonnet X, Girondot M, Prévot-Julliard AC (2008). Varying hydric conditions during incubation influence egg water exchange and hatchling phenotype in the red-eared slider turtle. Physiological and Biochemical Zoology.

[ref-6] Du WG, Hu LJ, Lu JL, Zhu LJ (2007). Effects of incubation temperature on embryonic development rate, sex ratio and post-hatching growth in the Chinese three-keeled pond turtle, *Chinemys reevesii*. Aquaculture.

[ref-7] Du WG, Ji X (2001). Influence of incubation temperature on embryonic use of material and energy in the Chinese soft-shelled turtle (*Pelodiscus sinensis*). Acta Zoologica Sinica.

[ref-8] Du WG, Ji X (2003). The effects of incubation thermal environments on size, locomotor performance and early growth of hatchling soft-shelled turtles, *Pelodiscus sinensis*. Journal of Thermal Biology.

[ref-9] Du WG, Shen JW (2007). Growth and sexual size dimorphism in the Chinese three-keeled pond turtle, *Chinemys reevesii*.

[ref-10] Du WG, Wang L, Shen JW (2010). Optimal temperatures for egg incubation in two Geoemydid turtles: *Ocadia sinensis* and *Mauremys mutica*. Aquaculture.

[ref-11] Du WG, Warner DA, Langkilde T, Robbins T, Shine R (2010). The physiological basis of geographic variation in rates of embryonic development within a widespread lizard species. American Naturalist.

[ref-12] Du WG, Zheng RQ (2004). Egg survival and hatchling traits of the Chinese three-keeled pond turtle (*Chinemys reevesii*) incubated in different hydric environments. Acta Zoologica Sinica.

[ref-13] Du WG, Zheng RQ, Shu L (2006). The influence of incubation temperature on morphology, locomotor performance, and cold tolerance of hatchling Chinese three-keeled pond turtles, *Chinemys reevesii*. Chelonian Conservation and Biology.

[ref-14] Finkler MS, Knickerbocker DL, Claussen DL (2000). Influence of hydric conditions during incubation and population on overland movement of neonatal snapping turtles. Journal of Herpetology.

[ref-15] Fordham DA, Georges A, Corey B (2007). Optimal conditions for egg storage, incubation and post-hatching growth for the freshwater turtle, *Chelodina rugosa*: science in support of an indigenous enterprise. Aquaculture.

[ref-16] Hernandez-Montoya V, Paez VP, Ceballos CP (2017). Effects of temperature on sex determination and embryonic development in the red-footed tortoise, *Chelonoidis carbonarius*. Chelonian Conservation and Biology.

[ref-17] Hewavisenthi S, Parmenter CJ (2001). Influence of incubation environment on the development of the flatback turtle (*Natator depressus*). Copeia.

[ref-18] Hou L (1985). Sex determination by temperature for incubation in *Mauremys reevesii*. Acta Herpetologica Sinica.

[ref-19] Janzen FJ, Morjan CL (2002). Egg size, incubation temperature, and posthatching growth in painted turtles (*Chrysemys picta*). Journal of Herpetology.

[ref-20] Ji X, Chen F, Du WG, Chen HL (2003). Incubation temperature affects hatchling growth but not sexual phenotype in the Chinese soft-shelled turtle, *Pelodiscus sinensis* (Trionychidae). Journal of Zoology.

[ref-21] Ji X, Zhang CH (2001). Effects of thermal and hydric environments on incubating eggs, hatching success, and hatchling traits in the Chinese skink (*Eumeces chinensis*). Acta Zoologica Sinica.

[ref-22] Jiang ZG, Jiang JP, Wang YZ, Zhang E, Zhang YY, Li LL, Xie F, Cai B, Cao L, Zheng GM, Dong L, Zhang ZW, Ding P, Luo ZH, Ding CQ, Ma ZJ, Tang SH, Cao WX, Li CW, Hu HJ, Ma Y, Wu Y, Wang YX, Zhou KY, Liu SY, Chen YY, Li JT, Feng ZJ, Wang Y, Wang B, Li C, Song XL, Cai L, Zang CX, Zeng Y, Meng ZB, Fang HX, Ping XG (2016). Red list of China’s vertebrates. Biodiversity Science.

[ref-23] Li GS (2005). The growth of hatchling turtles (*Chinemys reevesii*). Reservoir Fisheries.

[ref-24] Liu GA, Liu YQ, Hu DG, Xiong WH (1984). A preliminary observation on the development of the embryo of tortoise, *Chinemys reevesii*. Zoological Research.

[ref-25] Liu GA, Xu DY, Liu CS (1988). Studies on the reproductive ecology of the turtle, *Chinemys reevesii*. Acta Hydrobiology Sinica.

[ref-26] Miller K (1993). The improved performance of snapping turtles (*Chelydra serpentina*) hatched from eggs incubated on a wet substrate persists through the neonatal period. Journal of Herpetology.

[ref-27] Packard GC (1999). Water relations of chelonian eggs and embryos: is wetter better?. American Zoologist.

[ref-28] Packard GC, Packard MJ (2002). Wetness of the nest environment influences cardiac development in pre- and post-natal snapping turtles (*Chelydra serpentina*). Comparative Biochemistry and Physiology Part A: Molecular and Integrative Physiology.

[ref-29] Packard GC, Packard MJ, Miller K, Boardman TJ (1987). Influence of moisture, temperature, and substrate on snapping turtle eggs and embryos. Ecology.

[ref-30] Packard GC, Taigen TL, Packard MJ, Boardman TJ (1981). Changes in mass of eggs of softshell turtles (*Trionyx spiniferus*) incubated under hydric conditions simulating those of natural nests. Journal of Zoology.

[ref-31] Shi HT, Parham JF, Fan ZY, Hong ML, Yin F (2008). Evidence for the massive scale of turtle farming in China. Oryx.

[ref-32] Stubbs JL, Mitchell NJ (2018). The influence of temperature on embryonic respiration, growth, and sex determination in a western Australian population of green turtles (*Chelonia mydas*). Physiological and Biochemical Zoology.

[ref-33] Tucker JK, Filoramo NI, Paukstis GL, Janzen FJ (1998). Response of red-eared slider, *Trachemys scripta elegans*, eggs to slightly differing water potentials. Journal of Herpetology.

[ref-34] Tucker JK, Paukstis GL (2000). Hatching success of turtle eggs exposed to dry incubation environment. Journal of Herpetology.

[ref-35] Wang QD, Cheng L, Liu JS, Li ZJ, Xie SQ, De Silva SS (2015). Freshwater aquaculture in PR China: trends and prospects. Reviews in Aquaculture.

[ref-36] Wang L, Du WG, Shen JW, Zhu LJ (2010). Comparisons of egg incubation and hatchling traits among captive cohorts of the Chinese three-keeled pond turtle, *Chinemys reevesii*. Acta Ecologica Sinica.

[ref-37] Xiao FR, Shi HT, Sun L (2014). Sexual dimorphism in body size and shape in the four-eyed spotted turtle *Sacalia quadriocellata*. Chinese Journal of Zoology.

[ref-38] Yang ZC, Niu CJ, Sun RY (2002). Effects of temperature on egg incubation and embryo development of the soft-shelled turtle (*Trionyx sinensis*). Acta Zoologica Sinica.

[ref-39] Zhao B, Chen Y, Wang Y, Ding P, Du WG (2013). Does the hydric environment affect the incubation of small rigid-shelled turtle eggs?. Comparative Biochemistry and Physiology Part A: Molecular & Integrative Physiology.

[ref-40] Zhao WH, Zhu XP, Guo JH, Wei CQ, Chen YL (2009). Effects of different hydric environment on embryonic development and hatchling traits of yellow pond turtle (*Mauremys mutica* Cantor). Acta Ecologica Sinica.

[ref-41] Zheng RQ, Du WG, Zhang YP, Bao YX (2006). Influence of incubation temperature on embryonic use of energy and mineral metabolism in the Chinese three-keeled pond turtle *Chinemys reeresii*. Acta Zoologica Sinica.

[ref-42] Zhu XP, Wei CQ, Zhao WH, Du HJ, Chen YL, Gui JF (2006). Effects of incubation temperatures on embryonic development in the Asian yellow pond turtle. Aquaculture.

